# Short-Term Effects of Tirzepatide in Obese Adults: A Real-World Prospective Study

**DOI:** 10.7759/cureus.85970

**Published:** 2025-06-13

**Authors:** Nikos Adamidis, Athanasios Desalermos, Nektaria Papadopoulou, Sofia Adamidi, Theodoros Koutrakos, Maria Kyventidou, Vasiliki E Georgakopoulou, Sotirios Adamidis

**Affiliations:** 1 First Department of Internal Medicine, Sismanogleio Hospital, Athens, GRC; 2 Department of Gastroenterology, Athens Medical Group, Athens, GRC; 3 Department of Endocrinology, University Hospital of Linköping, Linköping, SWE; 4 Department of Internal Medicine, Charlton Memorial Hospital, Massachusetts, USA; 5 Department of Lifestyle Medicine, Athens Medical Group, Athens, GRC; 6 Department of Pathophysiology/Pulmonologist, Laiko General Hospital, Athens, GRC; 7 1st Department of Internal Medicine, Athens Medical Group, Athens, GRC

**Keywords:** body composition, fat mass, obesity, tirzepatide, weight loss

## Abstract

Background

Tirzepatide is a novel dual agonist of glucose-dependent insulinotropic polypeptide (GIP) and glucagon-like peptide-1 (GLP-1) receptors, shown to induce substantial weight loss in clinical trials. However, real-world data on the short-term effects of the drug in obese adults is unknown. This prospective observational study aimed to evaluate the short-term effects of tirzepatide on body composition-including fat mass, fat-free mass, total body water, and extracellular water-as well as waist circumference and body weight in adults with overweight or obesity under routine clinical care.

Methods

Adults with a body mass index (BMI) ≥27 kg/m² who were newly prescribed tirzepatide received once-weekly subcutaneous injections, alongside standardized lifestyle counseling. Body composition parameters, including fat mass (FM), fat-free mass (FFM), total body water (TBW), and extracellular water (ECW), were assessed using bioelectrical impedance analysis (BIA) under standardized conditions at baseline and after a median of 30 days. The study was conducted between December 1, 2024, and May 1, 2025. Changes from baseline were analyzed using the Wilcoxon signed-rank test; subgroup analyses were performed by sex and age. Spearman’s rank correlation coefficients were used to examine associations between age and treatment response.

Results

A total of 115 participants (67.5% female; median age 48 years) were included. Median body weight decreased by -4.0 kg, waist circumference by -5.0 cm, and fat mass by -3.6 kg. Changes in fat-free mass and total body water were minimal, suggesting preferential loss of adipose tissue. Although females demonstrated a slightly greater median reduction in body weight [median: -4.0 kg; interquartile range (IQR): -7.0 to -2.1 kg] compared to males (median: -3.0 kg; IQR: -6.0 to 0.0 kg), the difference was not statistically significant (p = 0.112). Similarly, no statistically significant differences were observed between sexes for the change in total body water [difference in TBW (DTBW): median for females: 0.1 L vs. -0.4 L for males, p = 0.733], extracellular water [difference in ECW (DECW): -0.6 L vs. 0.5 L, p = 0.240], or FFM [difference in FFM (DFFM): -0.7 kg vs. -0.6 kg, p = 0.276]. Age was not correlated with body composition changes.

Conclusion

Tirzepatide was associated with rapid and clinically meaningful reductions in body weight, central adiposity, and fat mass within 30 days, with preservation of lean mass, supporting its utility in early obesity management.

## Introduction

The rising prevalence of obesity has emerged as one of the most significant global health threats of the 21st century. In 2013, over 2.1 billion people were classified as overweight or obese, with numbers continuing to climb in both developed and developing nations [1]. Obesity is strongly associated with insulin resistance, type 2 diabetes mellitus (T2DM), dyslipidemia, hypertension, and an elevated risk of cardiovascular events and mortality [2]. Given its multifactorial etiology and the complex interplay of metabolic, behavioral, and genetic factors, the treatment of obesity remains a clinical challenge. Although lifestyle modifications and pharmacologic agents have formed the cornerstone of obesity management, the long-term efficacy of these interventions is often limited by weight regain and suboptimal adherence [3,4].

Tirzepatide is a novel dual agonist of glucose-dependent insulinotropic polypeptide (GIP) and glucagon-like peptide-1 (GLP-1) receptors. It represents a new class of incretin-based therapies that combines the glucose-lowering and weight-reducing benefits of both pathways [5]. GIP, traditionally thought to be less effective in patients with T2DM, has shown synergistic effects when co-activated with GLP-1, resulting in enhanced insulin secretion, delayed gastric emptying, improved satiety, and significant weight loss [6,7]. Clinical trials such as SURPASS and SURMOUNT have demonstrated that tirzepatide leads to reductions in body weight exceeding those achieved by selective GLP-1 receptor agonists, with a mean weight loss reaching up to 22.5% in individuals with obesity without diabetes [8,9]. These changes have also been accompanied by improvements in glycated hemoglobin (HbA1c), blood pressure, and lipid profiles, further supporting tirzepatide’s role as a comprehensive cardiometabolic agent [10].

Beyond overall weight loss, changes in body composition-particularly the reduction of visceral fat and the preservation of lean body mass-are crucial determinants of clinical benefit. Visceral adiposity is more strongly associated with insulin resistance, systemic inflammation, and cardiovascular disease than subcutaneous fat [11]. Moreover, excessive loss of fat-free mass (FFM), including muscle tissue, during pharmacologically induced weight loss may have adverse effects on metabolic health and functional capacity [12]. There is a growing interest in evaluating how emerging anti-obesity agents affect specific body indices, including fat mass (FM), FFM, total body water (TBW), and extracellular water (ECW), as well as anthropometric indicators such as waist circumference (WC), which serves as a surrogate for visceral adiposity [13,14].

Despite promising clinical trial results, there remains a gap in real-world data regarding the short-term physiological effects of tirzepatide, particularly on detailed body composition metrics. While tirzepatide has demonstrated substantial weight loss and metabolic improvements in randomized controlled trials, most studies have focused on long-term outcomes and used body weight as the primary endpoint. However, weight alone does not reflect qualitative changes in body composition-such as reductions in FM or preservation of lean tissue-which are critical for assessing the full metabolic benefits and sustainability of treatment. Therefore, real-world evidence examining tirzepatide’s effects on detailed body composition parameters over shorter periods is essential to inform clinical decisions and patient expectations.

The present prospective study aims to investigate the short-term effects of tirzepatide on detailed body composition parameters in adults with overweight or obesity under real-world clinical conditions. In addition to monitoring weight and WC, the study assesses changes in FM, FFM, TBW, and ECW to provide a comprehensive evaluation of tirzepatide’s physiological effects beyond the scale. These insights may help guide therapeutic decisions and optimize long-term outcomes in the pharmacological management of obesity.

## Materials and methods

Study design and participants

This was a prospective observational study conducted at Athens Medical Group between December 1, 2024, and May 1, 2025. Eligible participants were adults aged ≥18 years who were overweight and had comorbidities or obesity necessitating therapy [defined as body mass index (BMI) ≥27 kg/m² with comorbidities or ≥30 kg/m², respectively] [15], who were newly initiated on tirzepatide therapy for the management of excess body weight or obesity-related metabolic risk. Exclusion criteria included pregnancy, active malignancy, recent weight-loss surgery, or any condition affecting fluid balance or body composition (e.g., heart failure, nephrotic syndrome).

Intervention

All participants received subcutaneous tirzepatide injections once weekly. The starting dose was either 2.5 mg or 5 mg per week, titrated up to 5 mg over the first month of treatment based on tolerability and clinical response. Concurrently, participants were provided with standardized lifestyle advice, including dietary counseling based on Mediterranean diet principles and encouragement to engage in 45 minutes of brisk walking daily. More specifically, as part of the standardized clinical protocol, all participants received the same lifestyle guidance, which included: a) Diet: A Mediterranean-style dietary plan emphasizing high intake of vegetables, fruits, legumes, whole grains, and olive oil; moderate consumption of fish and poultry; and limited intake of red meat, processed foods, and refined sugars. Participants were advised to avoid calorie-dense snacks and sugary beverages, and b) Exercise: All participants were encouraged to engage in at least 45 minutes of brisk walking daily, five days per week.

Antihypertensive medications were continued as clinically indicated, with dose adjustments or discontinuation made in response to significant reductions in body weight or blood pressure.

Body composition measurements

Anthropometric and body composition measurements were performed at baseline (prior to initiation of tirzepatide) and at follow-up [median interval of 30 days; interquartile range (IQR) 21-55.75 days]. Weight and WC were measured using standard techniques with participants in light clothing and without shoes. Body composition parameters, including FM, FFM, TBW, and ECW, were assessed using bioelectrical impedance analysis (BIA) under standardized conditions (e.g., fasting state, no recent exercise, and no alcohol consumption within 24 hours). Measurements were conducted using the BIA 101 device (Akern Srl, Florence, Italy). Muscle mass was not reported separately in this study because the BIA 101 device used does not isolate skeletal muscle mass as an independent parameter; instead, muscle is incorporated within the broader category of fat-free mass, which also includes body water, bones, and organ tissue.

Data collection and outcomes

The primary outcomes were the absolute and relative changes in body weight, FM, FFM, TBW, ECW, and WC from baseline to follow-up. Secondary outcomes included the proportion of participants with clinically significant weight loss (defined as ≥5% of baseline body weight).

Statistical analysis

Continuous variables were tested for normality using the Kolmogorov-Smirnov test and found to be non-normally distributed; therefore, data are presented as medians with IQRs. Absolute changes between baseline and follow-up were calculated for key body composition parameters, including weight, WC, FM, FFM, TBW, and ECW. The Wilcoxon signed-rank test was used to assess within-subject changes over time. Sex-based comparisons of changes in body composition were performed using the Mann-Whitney U test. Associations between age and outcome variables were examined using Spearman’s rank correlation coefficients. A two-tailed p-value <0.05 was considered statistically significant. All statistical analyses were conducted using IBM SPSS Statistics for Windows, Version 29.0 (IBM Corp., Armonk, NY, USA).

## Results

Baseline characteristics and comorbidities

A total of 115 participants were enrolled in this study. The study population comprised predominantly female participants (67.5%), with a median age of 48.0 years (IQR: 41.0-56.0). The median body weight was 92.0 kg (IQR: 79.0-105.5), while the median WC was 97.5 cm (IQR: 85.75-115.5). Body composition analysis revealed a median FFM of 60.1 kg (IQR: 53.0-76.7), a median FM of 32.1 kg (IQR: 19.15-42.35), a median TBW of 47.6 L (range: 31-83), and median ECW of 19.35 L (IQR: 15.8-22.8). The median follow-up period was 30.0 days (IQR: 21.0-55.75).

Regarding comorbidities, the most frequently observed conditions included insulin resistance (98.2%), liver disease (89.5%), hyperlipidemia (86.0%), and arterial hypertension (71.9%). Lower prevalence rates were noted for diabetes mellitus (33.3%), respiratory disease (22.8%), coronary artery disease (14.9%), and other cardiac diseases (8.8%). Less common conditions included renal disease (7.9%), myocardial infarction (3.5%), and immunosuppression (2.6%), while no participants had a history of cerebrovascular disease.

The baseline characteristics of the study population are displayed in Table 1.

**Table 1:** Baseline Characteristics of the study population. ΒΜΙ: Body Mass Index; CAD: Coronary Artery Disease; ECW: Extracellular Water; FFM: Fat-Free Mass; IQR Interquartile Range; MI: Myocardial Infarction; TBW: Total Body Water

Overweight Group		
Characteristic	Median	IQR (25th–75th percentile)
BMI (kg/m^2^)	25.20	22.80–28.00
Weight (kg)	72.00	67.00–78.00
Age (years)	47.00	39.50–53.50
TBW (L)	41.90	36.20–46.60
ECW (L)	16.65	12.40–19.27
FFM (kg)	56.50	46.80–62.00
Characteristic	N	Percentage (%)
Female sex	32	97.0
Diabetes mellitus	3	9.1
Arterial hypertension	15	45.5
Coronary artery disease (CAD)	0	0.0
Myocardial infarction (MI)	0	0.0
Hyperlipidemia	21	63.6
Renal disease	0	0.0
Respiratory disease	3	9.1
Liver disease	23	69.7
Cerebrovascular disease	0	0.0
Immunosuppression	0	0.0
Other cardiac diseases	0	0.0
Insulin resistance	31	93.9
Obese Group		
Characteristic	Median	IQR (25th–75th percentile)
BMI (kg/m^2^)	33.45	31.60–37.98
Weight (kg)	100.00	90.00–114.00
Age (years)	48.00	42.00–58.50
TBW (L)	49.50	44.60–61.35
ECW (L)	20.95	16.83–23.78
FFM (kg)	61.55	55.65–80.68
Characteristic	N	Percentage (%)
Female sex	45	55.6
Diabetes mellitus	35	43.2
Arterial hypertension	67	82.7
Coronary artery disease (CAD)	17	21.0
Myocardial infarction (MI)	4	4.9
Hyperlipidemia	77	95.1
Renal disease	9	11.1
Respiratory disease	23	28.4
Liver disease	79	97.5
Cerebrovascular disease	0	0.0
Immunosuppression	3	3.7
Other cardiac diseases	10	12.3
Insulin resistance	81	100.0

Changes in body composition following tirzepatide treatment

Following treatment with tirzepatide, significant changes in body composition were observed. The median reduction in body weight was -4.0 kg (IQR: -7.0 to -2.1), with a maximum loss of -32.0 kg. WC showed a median decrease of -5.0 cm (IQR: -11.0 to 0.0). 

In terms of fluid compartments, TBW demonstrated a median change of -0.8 L (IQR: -2.9 to 2.2), while ECW decreased by a median of -0.6 L (IQR: -2.6 to 1.22). FFM showed a median decrease of -0.7 kg (IQR: -3.4 to 3.0), with a broad range of variation (-102.9 to 20.4 kg), indicating heterogeneous responses.

The reduction in FM was more consistent, with a median decrease of -3.6 kg (IQR: -7.02 to -2.4), based on a subset of 48 participants. Additionally, the median percentage change in lean mass relative to total weight loss was -4.65% (IQR: -6.96% to -2.85%), suggesting that most of the weight loss was attributable to FM reduction rather than lean tissue.

**Figure 1:**
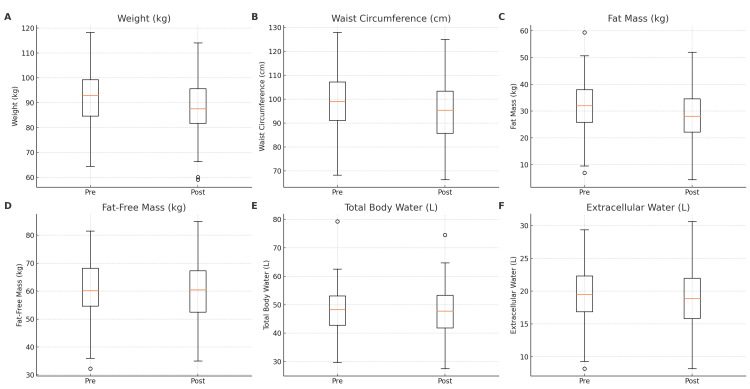
Short-term changes in body composition parameters following tirzepatide treatment. Boxplots display pre- and post-treatment values for key anthropometric and body composition measures: (A) Body weight (kg), (B) Waist circumference (cm), (C) Fat mass (kg), (D) Fat-free mass (kg), (E) Total body water (L), and (F) Extracellular water (L). Each panel shows the median (horizontal line), interquartile range (box), and outliers (dots).

The changes in body composition followιng tirzepatide treatment are displayed in Table 2 and Figure 1.

**Table 2:** Changes in Body Composition After Tirzepatide. D: difference; ECW: Extracellular Water; FM: Fat Mass; FFM: Fat-Free Mass; IQR Interquartile Range; TBW: Total Body Water

Variable	N	Median	Min	Max	IQR (25–75th percentile)
D Weight (kg)	113	-4.0	-32.0	-1.0	-7.0 – -2.1
D Waist Circ. (cm)	113	-5.0	-56.0	277.0	-11.0 – 0.0
D TBW (L)	83	-0.8	-19.2	22.8	-2.9 – 2.2
D ECW (L)	80	-0.6	-16.8	18.7	-2.6 – 1.22
D FFM (kg)	83	-0.7	-102.9	20.4	-3.4 – 3.0
D FM (kg)	48	-3.6	-16.6	-0.4	-7.02 – -2.4
D Weight (%)	113	-4.65	-25.4	-0.97	-6.96 – -2.85

Sex- and age- based differences in changes of body composition parameters

To further explore the influence of sex on body composition changes after tirzepatide administration, we performed descriptive analyses and non-parametric comparisons between male and female participants. Although females demonstrated a slightly greater median reduction in body weight (median: -4.0 kg; IQR: -7.0 to -2.1 kg) compared to males (median: -3.0 kg; IQR: -6.0 to 0.0 kg), the difference was not statistically significant (p = 0.112). Similarly, no statistically significant differences were observed between sexes for the change in total body water [difference in TBW (DTBW): median for females: 0.1 L vs. -0.4 L for males, p = 0.733], extracellular water [difference in ECW (DECW): -0.6 L vs. 0.5 L, p = 0.240], or FFM [difference in FFM (DFFM): -0.7 kg vs. -0.6 kg, p = 0.276] (Table 3).

**Table 3:** Gender-Based Changes in Body Composition. D: Difference; ECW: Extracellular Water; FM: Fat Mass; FFM: Fat-Free Mass; IQR Interquartile Range; TBW: Total Body Water

Variable	Female (Median [IQR])	Male (Median [IQR])	p-value
D Weight (kg)	-4.0 [–7.0 to –2.1]	-5.0 [–11.0 to –2.4]	0.112
D Waist Circ. (cm)	0.0 [–11.0 to 0.0]	-1.0 [–7.0 to –1.0]	0.567
D TBW (L)	1.0 [4.70]	-0.4 [7.25]	0.733
D ECW (L)	-0.6 [3.40]	0.5 [8.50]	0.240
D FFM (kg)	0.4 [8.50]	-0.6 [6.45]	0.276
D FM (kg)	-3.6 [4.50]	-3.0 [4.30]	0.268
D Weight (%)	-0.04 [0.02]	-0.03 [0.06]	0.112

Spearman’s correlation coefficients were calculated to investigate relationships between age and body composition changes. No significant associations were found between age and difference in weight (DWeight) (rho = 0.055, p = 0.562) or any other parameter, indicating that the effects of tirzepatide were independent of age (Table 4).

**Table 4:** Spearman’s Correlation Between Age and Changes in Body Composition Parameters. D: Difference

Parameter	Spearman’s rho	p-value
DWeight (kg)	0.055	0.562
DWaist Circumference (cm)	−0.043	0.643
DFat Mass (kg)	−0.072	0.491
DFat-Free Mass (kg)	0.027	0.755
DTotal Body Water (L)	0.016	0.844
DExtracellular Water (L)	0.008	0.913

## Discussion

In this real-world, 30-day prospective evaluation of tirzepatide in adults with overweight or obesity, we observed rapid and clinically meaningful reductions in body weight and fat mass, accompanied by minimal lean mass loss. All participants received standardized lifestyle counseling. The dietary intervention followed Mediterranean diet principles, with an emphasis on plant-based foods, olive oil, fish, and reduced intake of red meat and processed foods. Participants were also advised to engage in 45 minutes of brisk walking at least five times per week. These interventions were consistently applied across the cohort. The median weight loss was 4.0 kg, and the median fat mass (FM) reduction was 3.6 kg, indicating that most of the weight loss was attributable to adipose tissue. Waist circumference also decreased significantly, reflecting an early reduction in central adiposity. Importantly, treatment effects were consistent across age and sex, mirroring subgroup findings from pivotal tirzepatide trials that demonstrated uniform efficacy across demographic groups [16].

These short-term results align with the early phase of weight loss trajectories seen in randomized trials and real-world studies of tirzepatide. In the SURMOUNT-1 trial, a 72-week phase three study in people with obesity-tirzepatide 15 mg resulted in a ~21% reduction in TBW [16]. The parameters evaluated included body weight, WC, FM, FFM, TBW, and ECW, measured using multifrequency BIA. Body composition analysis from that trial showed that approximately three-quarters of the weight lost was FM, with lean mass loss comprising only ~10-11% of total reduction [16]. This fat-to-lean mass loss ratio appears more favorable than that reported in semaglutide trials, where lean mass contributed to ~39-40% of total weight loss [17]. These differences may reflect tirzepatide’s dual GIP/GLP-1 agonism and suggest a relative advantage in preserving lean tissue, although direct head-to-head body composition comparisons remain limited.

Real-world data are beginning to confirm tirzepatide’s high efficacy beyond controlled trial settings [18, 19]. A large retrospective study of over 4,000 individuals without diabetes showed that those who remained on tirzepatide for at least six months lost 12-13% of their body weight, despite often using lower doses than those used in trials [18]. Furthermore, head-to-head observational data have shown that tirzepatide induces greater weight loss than semaglutide when used for obesity. In a propensity-matched cohort study, weight loss at three months was 5.9% with tirzepatide vs. 3.6% with semaglutide, and at six months, 10.1% vs. 5.9%, respectively. By 12 months, tirzepatide users had lost ~15%, nearly double the reduction achieved with semaglutide (8%) [19].

In this context, our observation of ~4 kg weight loss over 30 days-equating to approximately 3-5% of baseline weight-is noteworthy. This degree of early response meets the commonly accepted ≥5% clinical benefit threshold and typically exceeds the short-term impact of lifestyle interventions alone. In SURMOUNT-1, early reductions were seen within the first few weeks of treatment initiation [16], and our findings parallel this rapid trajectory. While GLP-1 RAs such as semaglutide also induce early weight loss, their effects are often slower during the initial dose-escalation phase [17]. Tirzepatide’s dual incretin activity may contribute to its more robust early impact.

The quality of the weight loss observed, predominantly fat rather than lean mass-is clinically important. Rapid reductions in FM, particularly with accompanying decreases in waist circumference, are suggestive of a decline in visceral adiposity, which is closely linked to insulin resistance and cardiovascular risk. Longer-term tirzepatide trials have demonstrated a ~40% reduction in visceral fat area over 72 weeks [16]. It is therefore plausible that early therapy, even before large-scale weight loss is achieved, may confer metabolic improvements such as enhanced insulin sensitivity or lower blood pressure. In clinical practice, these early benefits may positively influence patient motivation and treatment adherence.

Preserving lean mass during weight loss is equally critical for maintaining physical function and metabolic health. Excessive muscle loss can impair strength, reduce resting metabolic rate, and increase the risk of frailty or weight regain. Our finding of minimal lean mass loss during this early treatment phase is encouraging and supports the idea that tirzepatide promotes metabolically beneficial, high-quality weight loss. These observations are consistent with literature suggesting that GLP-1-based therapies do not disproportionately deplete muscle mass relative to overall weight loss [20].

This study has several limitations that should be acknowledged. First, as a real-world, observational study without a control group, causality cannot be established. The observed reductions in weight and fat mass cannot be attributed solely to tirzepatide, as concurrent lifestyle interventions-including standardized dietary counseling and encouragement of physical activity-may have contributed to the results. However, the lack of detailed data on adherence to lifestyle recommendations limits our ability to disentangle their specific effects. Second, the study population was not stratified into overweight and obese subgroups, despite their likely differing responses to treatment. Given the potential for metabolic and physiological heterogeneity between these two groups, this limits the granularity of our findings. Future studies with larger cohorts should incorporate BMI-based subgroup analyses to better characterize response profiles. Third, the follow-up period was relatively short (median 30 days) and captured only the initial phase of treatment, when most participants were still undergoing dose escalation. Therefore, the findings represent an early snapshot rather than sustained outcomes. Long-term data are needed to assess whether the favorable fat-to-lean mass loss ratio persists and how it translates into cardiometabolic benefits. Fourth, body composition was assessed using bioelectrical impedance analysis (BIA), a practical but less accurate method compared to gold-standard techniques such as dual-energy X-ray absorptiometry (DEXA) or MRI. BIA is also sensitive to hydration status, which may affect estimates of fat-free and fat mass. Although standard measurement conditions were followed, rapid fluid shifts during early weight loss could introduce bias. Fifth, while fat mass reduction was observed in a subset of participants, full-body composition data were not available for the entire cohort, which may limit generalizability. Furthermore, sample size limitations reduced statistical power for subgroup analyses, potentially masking subtle sex- or age-specific differences. Finally, although the study shows minimal early lean mass loss, the potential for muscle wasting and sarcopenia remains a concern, particularly in the context of longer-term pharmacotherapy and in vulnerable populations. Future research should incorporate functional measures (e.g., handgrip strength, gait speed) and investigate adjunctive strategies such as resistance training and high-protein diets to safeguard muscle mass.

## Conclusions

Short-term tirzepatide treatment led to rapid and clinically significant reductions in body weight, fat mass, and waist circumference within 30 days, with minimal lean mass loss. These effects were consistent across sexes and age groups, supporting its early utility in obesity management. Given the predominance of fat over lean tissue loss, tirzepatide may offer a qualitatively superior weight loss profile. While longer-term outcomes remain to be confirmed, early incorporation of resistance training and protein-rich diets may help optimize muscle preservation during treatment.
